# Frailty Phenotype and Healthcare Costs in Women in Late Life: The SOF study

**DOI:** 10.1093/geroni/igaa057.2814

**Published:** 2020-12-16

**Authors:** Lisa Langsetmo, Allyson Kats, John Schousboe, Tien Vo, Brent Taylor, Kristine Ensrud

**Affiliations:** 1 University of Minnesota, Minneapolis, Minnesota, United States; 2 University of Minnesota, St Louis Park, Minnesota, United States

## Abstract

We used data from 1324 women (mean age 83) at the 2002-2004 exam linked with their Medicare claims to determine the association of the frailty phenotype with healthcare costs. The frailty phenotype was categorized as robust, pre-frail or frail. Multimorbidity and a frailty indicator (approximating the deficit accumulation index) were derived from claims. Functional limitations were assessed by asking about difficulty performing IADL. Total direct healthcare costs were ascertained during 36 months following the exam. Compared with robust, pre-frailty and frailty were associated with higher costs after accounting for demographics, multimorbidity, functional limitations and the frailty indicator (cost ratio 1.37 [1.10-1.71] among pre-frail and 1.63 [1.28-2.08] among frail). Discrimination of high-cost (top decile) women was improved by adding the phenotype and functional limitations to a model containing demographics and the claims-based measures. Findings suggest that assessment of the phenotype may improve identification of individuals at higher risk of costly care.

